# A Giant Mucinous Adenocarcinoma Arising within a Villous Adenoma of the Urachus: Case Report and Review of the Literature

**DOI:** 10.1155/2009/818646

**Published:** 2010-02-16

**Authors:** Steven Joniau, Evelyne Lerut, Hein Van Poppel

**Affiliations:** ^1^Department of Urology, University Hospitals Leuven, Herestraat 49, 3000 Leuven, Belgium; ^2^Department of Pathology, University Hospitals Leuven, Herestraat 49, 3000 Leuven, Belgium

## Abstract

We present an exceptional case of a giant urachal tumor, consisting of both villous adenoma and mucinous adenocarcinoma of the urachus. The tumor was incidentally discovered during investigations for renal failure. Initial transurethral biopsies showed only a villous adenoma of the urachus. Although the biopsies showed no malignancy, a radical cystoprostatectomy and broad excision of the urachus and umbilicus were performed. At the same time, a bilateral nephroureterectomy was performed because of reflux-nephropathy and renal failure. The indication for surgery was based on the typical imaging aspects, raising the suspicion of an underlying urachal adenocarcinoma (size and location). Indeed, at final histopathology a concomitant well-differentiated adenocarcinoma of the urachus confined to the urachal mucosa was found. The patient remained free of disease for 50 months of follow-up. Only three previous cases of urachal adenocarcinoma associated with villous adenoma have been described.

## 1. Introduction

Primary adenocarcinoma of the bladder arises from the urachal remnants or from the bladder urothelium. This entity represents no more than 0.5%–2% of all malignant bladder tumors. Urachal adenocarcinoma is extremely uncommon and accounts for 0.11%–0.34% of all bladder tumors. Intestinal metaplasia of the urachal epithelium is the most frequent mechanism accounting for the majority of adenocarcinomas of the urachus. Tumors of the urachal remnant originate from the juxtavesical segment of the urachus. While growing, they start to protrude in the bladder dome. Most frequently, they present with haematuria, pain, irritative voiding symptoms, and a lower midline abdominal mass. In some patients, mucousuria may be within the presenting symptoms [1, 2]. 

A urachal tumor is prone to extravesical growth and has a poor prognosis [3]. Radical or partial cystectomy is the treatment of choice, including wide resection of the umbilicus and the urachal tumor [1, 3]. 

Villous adenoma is not frequently found outside the large bowel. In the literature, less than 30 cases of villous adenoma of the urachus have been described [4–7]. We describe a case of a urachal adenocarcinoma arising within in a villous adenoma of the bladder. Only three similar cases have been described in the literature [8–10].

## 2. Case Report

**Figure 1:**
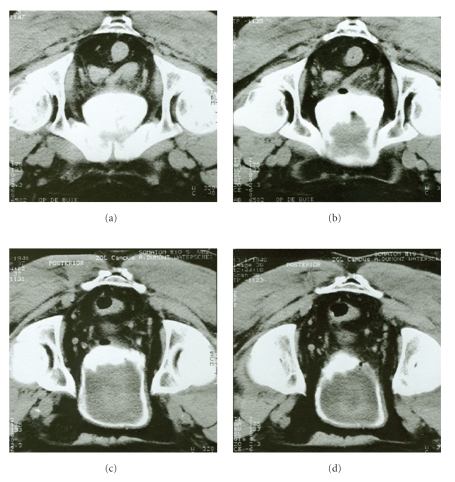
Contrast enhanced CT-scan of the abdomen (prone position), showing a large bladder mass, protruding the bladder and almost completely filling up the bladder lumen.

A 60-year-old man was referred to our institution because of a sudden decline in renal function. He had a medical history of chronic renal failure as a result of bilateral vesicoureteral reflux nephropathy. Apart from nocturia and urgency, he had no complaints. Physical examination was unremarkable. Lab results revealed a serum Creatinin of 4.19 mg/dL, a Creatinin clearance of 22 mL/min, and a proteinuria of 3.48 g/24h. Urinalysis showed no abnormalities. An abdominal ultrasound showed a polypoid, irregular mass, almost completely replacing the bladder lumen. A cystoscopy demonstrated a mucinous, nearly transparent bladder mass with prominent exophytic growth, which inserted at the dome of the bladder. Transurethral tumor biopsies showed a villous adenoma. Malignant cells could not be found in the biopsies. Because of the tumor size, no attempt was made to endoscopically resect the tumor. A subsequent CT-scan of the abdomen and pelvis confirmed a tumoral mass originating from the bladder dome. No invasion of the perivesical tissue was noticed (Figure 1). 

No pelvic or abdominal adenopathies were found. Both kidneys showed extensive cortical damage, with loss of nearly all the cortex of the right kidney. There was a prominent bilateral hydro-ureteronephrosis. The size and location of the tumor raised the suspicion for an underlying urachal adenocarcinoma, although no malignant cells were found in the biopsies. Since the kidney function was rapidly deteriorating and both kidneys showed extensive cortical loss, we decided to perform a radical cystoprostatectomy with bilateral nephroureterectomy. A urinary conduit was installed in view of a future renal transplant.

The postoperative course was uneventful. The patient was started on haemodialysis. He was discharged from our hospitals two weeks after the operation and has been without disease recurrence for 50 months.

## 3. Histopathology

**Figure 2:**
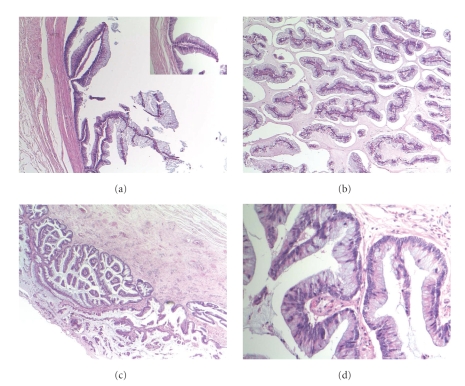
(a) Villous adenoma of the bladder with complex branching papillary structures, that are distended due to the presence of the exophytic mass in the lumen. There is no invasion into the underlying bladder wall ‘ (Hematoxylin Eosin stain, original magnification 50×) Inset: Higher magnification shows that the fibrovascular stalks of the adenoma are lined by pseudostratified mucinous epithelium with only mild degree of anisokaryosis. (Hematoxylin Eosin stain, original magnification 200×), (b) Urachal mucinous adenocarcinoma characterized by small sheets and nests of columnar mucinous cells floating in extracellular mucin. (Hematoxylin Eosin stain, original magnification 50×), (c) The cysts are lined with urachal mucinous adenocarcinoma with invasion in the bladder wall. (Hematoxylin Eosin stain, original magnification 25×), (d) Detail of papillary projections coated with columnar mucinous cells with pseudostratification, nuclear hyperchromasia and crowding, and goblet cells. (Hematoxylin Eosin stain, original magnification 200×).

Macroscopic examination showed the presence of a mucinous mass with a fibrous stalk of 2.5 cm emerging from the bladder dome, identified as being the urachus. The mass weighed 40 gr and was 16 cm long. In the centre of the mass, cysts filled with mucin were discovered. They were lined with urachal mucinous adenocarcinoma with beginning invasion into the underlying bladder wall. Overall, the diagnosis of a well-differentiated adenocarcinoma of the urachus, confined to the urachal mucosa (Sheldon stage I), was made [1]. Nearby, villous adenoma of the bladder was seen, appearing as complex branching papillary structures lined by a pseudostratified mucinous epithelium. (Figures 2(a)–2(d)).

## 4. Discussion

The urachus is a vestigial remnant of the allantois that normally obliterates during the descent of the bladder into the pelvis [1]. These urachal remnants are the source of glandular epithelium, derived by metaplasia according to the totipotent cell theory of Mostofi et al., and can undergo malignant transformation [2]. 

Urachal neoplasms can be benign (adenoma) or malignant. The *benign* adenomas are cystic tumors lined by mucus secreting colonic or rectal type epithelium [4]. Villous adenomas have been reported as papillary enteric adenoma or villous tumor. The origin may be from the urachus or caused by chronic irritation. Villous adenomas of the bladder are rare with less than 30 cases described [4–10]. Some cases were coincident with cystitis glandularis, or with adenocarcinoma, or with squamous cell carcinoma. The mean age at presentation was ≈65 years. There seems to be a link between villous adenomas, chronic irritation, and cystitis glandularis [7]. Expression of p53 in villous adenoma, but not in areas of cystitis glandularis, suggests that genetic progression along the lines of the colonic adenoma-carcinoma sequence may be responsible for the development of villous adenoma in the bladder. A thorough follow-up of all patients is recommended, considering that this condition may be premalignant [6–10]. The present case indeed clearly shows that a villous adenoma can further develop to an adenocarcinoma, and allows us to better understand the premalignant potential of such lesions.

Urachal *malignant* tumors have been classified as mucin-positive adenocarcinoma (69%), mucin-negative adenocarcinoma (15%), sarcoma (8%), squamous cell cancer (3%), transitional cell carcinoma (3%), and others (2%) [1]. A urachal malignant tumor tends to occur at the junction of the urachus and the bladder. The origin in the bladder dome, the presence of an urachal remnant, the sharp demarcation between tumor and normal urothelium, and the absence of cystitis glandularis and cystitis cystica (which can predispose to primary adenocarcinoma of the bladder) are the diagnostic criteria for a urachal remnant carcinoma [2, 3].

The majority of the urachal adenocarcinomas occur in the fifth and sixth decades, with a male/female ratio of 2.8/1. Haematuria, mucousuria, suprapubic pain or mass are frequently mentioned as first symptoms. Nevertheless, symptoms may be nonspecific irritative urinary symptoms. Whenever a bladder dome lesion presents with haematuria or mucousuria, a urachal adenocarcinoma must be considered, even if transurethral biopsies show no malignancy as in the presented case. A palpable midline infraumbilical mass on clinical examination is uncommon.

Diagnosis is mainly based on imaging and cystoscopy. Abdominal ultrasound may demonstrate a mass lesion in the dome of the bladder. CT-scan is the most sensitive imaging tool in diagnosing and staging of urachal tumors. It allows evaluation of local tumor extension and of local and distant lymph node involvement. Urinalysis usually reveals haematuria, pyuria, and mucousuria. Urine cytology has a high incidence of false negative results. A cystoscopy with or without tumor biopsy will usually provide the final diagnosis of the presence of a dome lesion but will give no information on the degree of malignancy of the lesion.

The treatment of urachal adenocarcinoma is still subject to debate. The number of patients reported in the literature is small and the biologic behaviour of these tumors still remains ill defined. Radical or partial cystectomy, both with broad excision of the umbilicus and urachal remnant, is the treatment of choice [1, 3]. Outcome seems to be more related to the stage of the disease than to the extent of the surgery. Less aggressive surgery can be recommended for well-differentiated colonic-type tumors. Without bladder epithelium invasion, urachal tumors usually tend to grow towards the bladder lumen and leave a sharp demarcation between tumor and normal bladder wall, thus allowing resection of the tumor with a broad margin of normal bladder tissue [3]. Sheldon et al. showed good results with combined extended partial cystectomy and urachectomy for well-selected patients [1]. 

Once a urachal tumor grows extra-vesically, it has a poor prognosis. Local recurrences generally cause death despite of salvage procedures [3]. Recurrence is usually seen in the first two postoperative years. The most common sites of recurrence are the pelvis, the bladder, the wound, or the abdominal wall [1]. 

In the present case, urachal adenocarcinoma was coexistent with villous adenoma, adding evidence to the theory that adenocarcinoma can develop along the adenoma-villous adenoma-adenocarcinoma line. To the best of our knowledge, only 3 cases of coexistent villous adenoma and adenocarcinoma of the urachus have been described so far [8–10]. Villous adenoma of the urachus should be considered a premalignant lesion and should be treated aggressively.

## 5. Synopsis

Adenocarcinoma of the urachus is an infrequent, but very aggressive type of bladder cancer. In localized disease, surgery is the mainstay of treatment. Less is known about precursors of this aggressive cancer. Villous adenoma is a well-recognized premalignant lesion in the colon. We provide evidence that this same lesion, if originating from the urachus, can be considered a precursor of adenocarcinoma of the urachus, and should be treated aggressively.
